# From gut to brain: unveiling probiotic effects through a neuroimaging perspective—A systematic review of randomized controlled trials

**DOI:** 10.3389/fnut.2024.1446854

**Published:** 2024-09-18

**Authors:** Annachiara Crocetta, Donato Liloia, Tommaso Costa, Sergio Duca, Franco Cauda, Jordi Manuello

**Affiliations:** ^1^Department of Psychology, Functional Neuroimaging and Complex Neural Systems (FOCUS) Laboratory, University of Turin, Turin, Italy; ^2^Department of Psychology, GCS fMRI, Koelliker Hospital, University of Turin, Turin, Italy; ^3^Neuroscience Institute of Turin (NIT), University of Turin, Turin, Italy; ^4^Move’N’Brains Lab, Department of Psychology, University of Turin, Turin, Italy

**Keywords:** probiotics, gut–brain axis, fMRI, neuroimaging, gut microbiota, depression, IBS

## Abstract

The gut–brain axis, a bidirectional communication network between the gastrointestinal system and the brain, significantly influences mental health and behavior. Probiotics, live microorganisms conferring health benefits, have garnered attention for their potential to modulate this axis. However, their effects on brain function through gut microbiota modulation remain controversial. This systematic review examines the effects of probiotics on brain activity and functioning, focusing on randomized controlled trials using both resting-state and task-based functional magnetic resonance imaging (fMRI) methodologies. Studies investigating probiotic effects on brain activity in healthy individuals and clinical populations (i.e., major depressive disorder and irritable bowel syndrome) were identified. In healthy individuals, task-based fMRI studies indicated that probiotics modulate brain activity related to emotional regulation and cognitive processing, particularly in high-order areas such as the amygdala, precuneus, and orbitofrontal cortex. Resting-state fMRI studies revealed changes in connectivity patterns, such as increased activation in the Salience Network and reduced activity in the Default Mode Network. In clinical populations, task-based fMRI studies showed that probiotics could normalize brain function in patients with major depressive disorder and irritable bowel syndrome. Resting-state fMRI studies further suggested improved connectivity in mood-regulating networks, specifically in the subcallosal cortex, amygdala and hippocampus. Despite promising findings, methodological variability and limited sample sizes emphasize the need for rigorous, longitudinal research to clarify the beneficial effects of probiotics on the gut–brain axis and mental health.

## 1 Introduction

The complex relationship between the gut and the brain, known as the gut–brain axis, has increasingly gained significant attention in scientific research. This has led to a reassessment of our understanding of how the digestive system and mental health interact. As interest in this domain expands, researchers are also exploring the role of probiotics in modulating this gut–brain interaction (1–3). Probiotics, beneficial microorganisms, are thought to potentially stabilize gut health and, by extension, affect neurological and psychological outcomes (4, 5). In addition, the development of neuroimaging techniques like functional magnetic resonance imaging (fMRI) has enabled researchers to directly investigate brain activity in response to alterations in the gut microbiota, providing a clearer picture of the physiological underpinnings of this connection (6, 7). The present review delves into these key areas, exploring both the impact of probiotics on the gut–brain axis and the insights garnered from neuroimaging studies, which collectively broaden the understanding of the therapeutic potentials of targeting the gut–brain pathway.

### 1.1 Gut–brain axis: mechanisms of bidirectional communication

The gut–brain axis forms a complex network that includes the gut, the central and enteric nervous systems (ENS), and is facilitated by neurological, immunological, and endocrine pathways (8–10). Research reveals that gut microbiota can significantly influence brain function and behavior through mechanisms such as neurotransmitter production, inflammation modulation, and hormone regulation (7, 8, 11–13).

The ENS, often referred to as the “second brain,” is a distinct branch of the autonomic nervous system that extends throughout the gastrointestinal tract (14). This complex network of neurons and glia is permanently linked to the brain via both the vagus nerve and the extrinsic sympathetic nervous system. The vagus nerve, a critical component of this communication network, transmits peripheral immune signals and regulates functions such as mood, digestion, and immune response. Situated beneath the gut epithelium, it facilitates the relay of signals from the gastrointestinal tract to the central nervous system (CNS) via afferent nerves. This process translates gut sensory information into neural, hormonal, and immunological signals (10). The extrinsic sympathetic nervous system, particularly spinal afferent neurons with cell bodies in the dorsal root ganglia, also significantly contributes to the gut–brain communication pathway, encoding sensory stimuli into neural action potentials (15, 16).

Beyond neural pathways, the immune system also contributes through cytokines, and maintaining gut barrier integrity, which prevents harmful substances from entering the bloodstream and causing inflammation that can impact brain health (10). Moreover, endocrine signaling, particularly through the hypothalamic-pituitary-adrenal (HPA) axis, regulates stress hormones like cortisol, while gut bacteria influence neurotransmitter production, including serotonin and dopamine, which are critical for mood regulation and emotional well-being (8). The gut–brain axis not only plays a pivotal role in maintaining health but also in the pathogenesis of both gastrointestinal and neuropsychiatric disorders (17–19).

While the gut–brain axis is well-recognized for its bidirectional communication (9, 10), the majority of research has concentrated on how the gut influences the brain. However, it is noteworthy to note that even though fewer studies have explored the reverse—how the brain impacts gut functions (20–24)—there is interesting and significant evidence in this direction. Through the autonomic nervous system (ANS), the brain regulates crucial gut functions such as motility, secretion, and mucosal immunity, thereby modulating its composition and overall activity (23). Notably, stress disrupts the homeostasis of the body, affecting both physiological states and the microbial ecosystems within the gastrointestinal tract (20, 25, 26). This phenomenon has been demonstrated in studies using laboratory animal models, which provide evidence that maternal stress during fetal development impacts the gut microbiota and other critical physiological systems (27). Additionally, chronic stress experienced during early life and continuing into adulthood can lead to further dysregulation (27). Furthermore, stress and anxiety increase the production of noradrenaline and glucocorticoids, which impact brain-to-gut communication (28, 29).

On the other hand, therapeutic interventions like psychotherapy and mindfulness may also impact the gut microbiota, highlighting the potential for “brain-based” interventions to affect gastrointestinal health (20, 25, 30–32). Psychotherapeutic therapies seem to enhance the quality of life in patients with gastrointestinal disorder, such as inflammatory bowel disease (IBD), as measured by questionnaires (33). Moreover, recent research highlights the potential for identifying new biomarkers that can assess gut health in people with substance use disorders, utilizing brain-derived markers such as evoked potentials to monitor pain responses influenced by morphine-like drugs (34).

### 1.2 Probiotics

Another element that have the potential to impact on the gut–brain axis are probiotics, defined as live microorganisms that confer health benefits when consumed in adequate amounts and play a critical role in maintaining intestinal microbiota balance (35, 36). Oral consumption of probiotics can directly modify the gut microbiota by enhancing the variety and quantity of beneficial microorganisms. This might potentially result in changes in the synthesis of metabolites derived from the microbiota, reduction in inflammation, modifications to the function of the HPA axis, and variations to the integrity of the gut barrier (37, 38). Hence, through gut–brain axis, probiotics have shown efficacy in modulating gut microbiota in both healthy individuals and those with gastrointestinal conditions. But further, they offer the possibility to influence the CNS (7, 39) and this has led to the exploration of probiotics as therapeutic adjuncts in treating CNS disorders such as cognitive deficits and mental disorders in both clinical and experimental settings (8, 39). For instance, animal studies have demonstrated that probiotics can alleviate anxiety-like behaviors and modulate stress responses, possibly through vagal pathways influencing brain regions associated with stress and anxiety (40). Similarly, in human trials, probiotics have shown promise in reducing psychological symptoms such as anxiety and depression, further supporting the therapeutic potential of modulating the gut microbiota in improving mental health and quality of life in various populations (41). Despite this, many assessments in these trials relied solely on self-reported measures, limiting the definitive conclusions that can be drawn (33).

### 1.3 Neuroimaging approach

Neuroimaging techniques, such as functional MRI (fMRI), provide an objective and detailed method for observing brain activity in response to probiotic intake. fMRI works by detecting changes in blood oxygen levels, which correlate with neural activity, allowing researchers to visualize and measure brain function in real time (42, 43). This method strengthens the investigation by revealing different aspects and addressing the limitations of relying solely on questionnaires to study the effects of probiotics.

The technology and protocols of fMRI have seen three decades of intense development, providing an unprecedented tool for *in-vivo* assessment of the neurophysiological basis of various conditions (44, 45). Functional MRI can be divided into two main types: task-based fMRI and resting-state fMRI. Task-based fMRI involves participants performing specific tasks while their brain activity is measured, providing insights into brain function related to cognitive and motor tasks (46). In contrast, resting-state fMRI measures brain activity when a person is not performing any explicit tasks, capturing the brain’s intrinsic functional connectivity (47). Resting-state fMRI has proven valuable in clinical settings, especially for presurgical mapping and in cases where patients cannot perform tasks due to age or cognitive impairments (48). This method allows for the identification of functional networks, such as the default mode network, salience network, and executive control network, which are critical for understanding the baseline functional architecture of the brain (49).

By examining changes in brain activity patterns and functional connectivity, fMRI studies have begun to demonstrate how alterations in the gut microbiota can influence neural circuits associated with mood and cognition (50). It has been particularly useful in identifying brain function variations in gastrointestinal diseases like irritable bowel syndrome (IBS), as well as in healthy individuals before and after prolonged probiotic intake (51). The non-invasive nature of fMRI and its ability to precisely map brain activity make it an important tool to validate subjective reports and clinical observations, offering a more comprehensive understanding of the gut–brain interaction (52–55). Recent experimental designs combining fMRI with probiotic administration have shed light on the specific brain activity changes associated with gut microbiota alterations (56–58). These studies have shown that probiotics can alter brain activity, particularly in areas involved in emotional processing and stress response. However, there is the need for more rigorous and expansive research to firmly establish the causative links between probiotic intake, brain activity changes, and clinical outcomes.

The aim of this systematic review is to provide a comprehensive analysis of the existing literature that explores the possible relationship between probiotics, gut microbiota, and alterations in brain activity through fMRI, in both healthy and clinical individuals after probiotic consumption. Furthermore, this review seeks to assess the strengths and weaknesses of the methodology and emphasize areas that require further research to gain a deeper understanding of the underlying mechanisms. Specifically, the review will focus on the effects of probiotics on brain activity and connectivity as evidenced by fMRI, evaluate the relationship between changes in the gut microbiota and alterations in brain function following probiotic supplementation, and highlight future research directions to address existing gaps in the understanding of the gut–brain interaction.

## 2 Methods

### 2.1 Retrieval strategy

The following databases were searched until April 2024 for relevant RCTs: PubMed, Web of Science, Scopus, the Cochrane Central Register of Controlled Trials and ClinicalTrials.gov. Furthermore, reference lists of each included study were reviewed to determine whether there was any further relevant publication.

The search terms included were:

**Pubmed:** (“probiotics”) AND (“gut–brain axis” OR “microbiota-gut–brain axis”) AND (neuroimaging OR MRI OR fMRI OR “positron emission tomography”).

**Google scholar:** “probiotics” AND “gut–brain axis” AND (neuroimaging OR “magnetic resonance imaging” OR fMRI OR “positron emission tomography”).

**ScienceDirect (SCOPUS):** “probiotics supplementation neuroimaging.”

**The Cochrane Central Register of Controlled Trials:** “Probiotics and fMRI.”

**ClinicalTrials.gov:** “Probiotics and fMRI.”

### 2.2 Eligibility criteria

To ensure a rigorous and comprehensive examination of the effects of probiotics on brain function, this review focuses exclusively on randomized clinical controlled trials. These studies provide the most reliable evidence by comparing the outcomes of participants randomly assigned to either a probiotic intervention or a placebo control group (59–61). This approach has the potential to minimize possible bias and allows for a clearer understanding of the causal relationships between probiotic consumption and changes in brain activity. The participants included in the reviewed studies span both healthy individuals and those with clinical conditions. By encompassing a broad range of cohorts worldwide, the review aims to provide insights into how probiotics might influence brain function across different populations, including those with existing health conditions that could potentially benefit from such interventions. The primary focus of the intervention is the administration of probiotics, with the outcomes compared against those of a placebo group. This direct comparison helps to isolate the effects of probiotics from other variables that might influence brain function. The studies assessed in this review specifically measure functional changes in the brain using fMRI, both task-based and resting-state.

Non-randomized clinical controlled trials are excluded because they do not provide the same level of evidence as randomized trials (62–64). Additionally, editorials, literature reviews, and meta-analyses are not considered, as they do not present original research data. Duplicate publications are also excluded to prevent redundancy and ensure that each included study offers unique data and insights. Finally, studies that do not provide a full report of results or primary data are not included, as the lack of complete information hinders the ability to thoroughly assess the findings of study and related implications. In particular, to assess the quality of the included studies, we utilized the Critical Appraisal Skills Programme (CASP) checklist for Randomized Controlled Trials (RCTs). The CASP checklist is a standardized tool that evaluates the methodological quality of studies based on 11 key questions (Supplementary Table S1). These questions address the validity of the study design, the precision of the results, and the applicability of the findings to the local population (Supplementary Table S2).

### 2.3 Data extraction and study selection

Data were extracted from each eligible study, including the following information: authors, year of publication, sample size, demographic characteristics of the sample (i.e., age, ethnicity, sex), details about the intervention group and control/placebo group (e.g., type, duration, dose, and time points), the biological data analysis strategy used, the fMRI data analysis strategy used, and the outcomes and conclusions of the study. The demographic data and clinical conditions for each fMRI study are included in Table 1.

**Table 1:** Experiments included in the systematic review: sample size demographic data for each fMRI studies.

Author (Year)	Subjects	Study group		Mean Age (SD)	Female (%)
		Probiotic group	Placebo group		
Bagga et al. (2018) (56)	30	15	15	Range 20–40	NA
Bagga et al. (2019) (51)	30	15	15	26.24 (4.76)	23%
Carlman et al. (2022) (79)	22	11	11	24.2 (3.4)	72,72%
Michels et al. (2016) (80)	11	6	5	23.3 (3.6)	100%
Papalini et al. (2019) (57)	58	29	29	21.5 (2.44)	100%
Pinto-Sanchez et al. (2017) (101)	44	22	22	43.25 (8.5)	22,20%
Rode et al. (2022) (73)	22	11	11	24.2 (3.4)	62,50%
Rode et al. (2022) (73)	22	11	11	24.2 (3.4)	62,50%
Schaub et al. (2022) (102)	47	21	26	39.1 (10.88)	23,50%
Tillisch et al. (2013) (58)	33	22	11	30 (10.4)	100%
Yamanbaeva et al. (2023) (103)	32	14	18	37.6 (10.81)	57,93%

### 2.4 Registration and reporting of results

**Figure 1:**
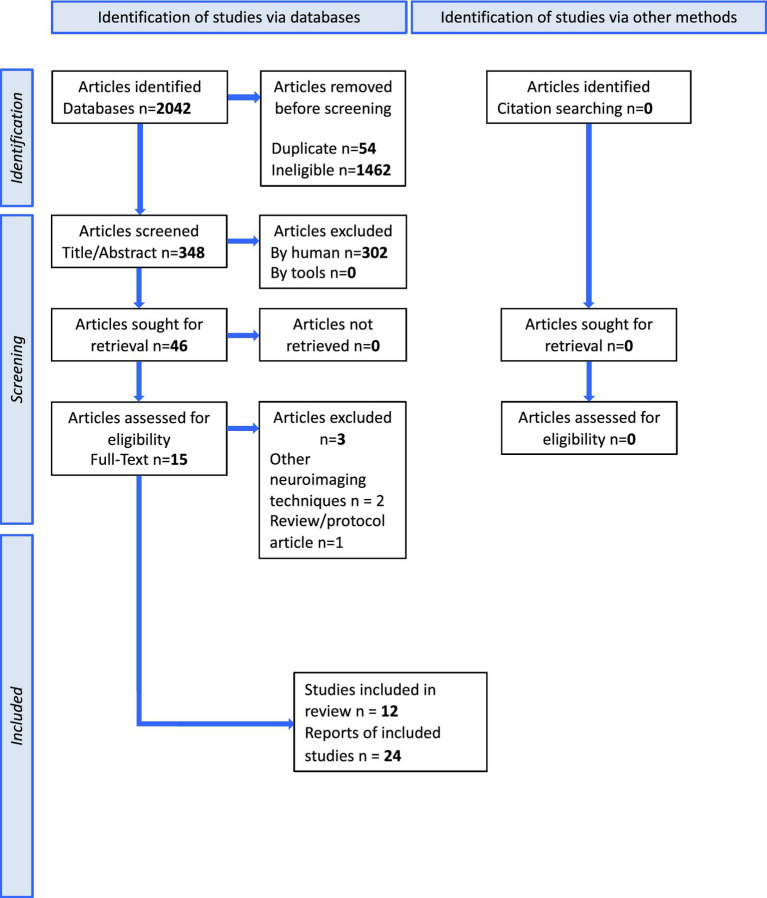
PRISMA flowchart for data selection.

This protocol was drafted according to the Preferred Reporting Items for Systematic Reviews and Meta-Analyses Protocols (PRISMA-P). The methods and results of the systematic review will be reported in accordance with the PRISMA-P guidelines (Figure 1).

## 3 Results

### 3.1 Study characteristics

**Figure 2:**
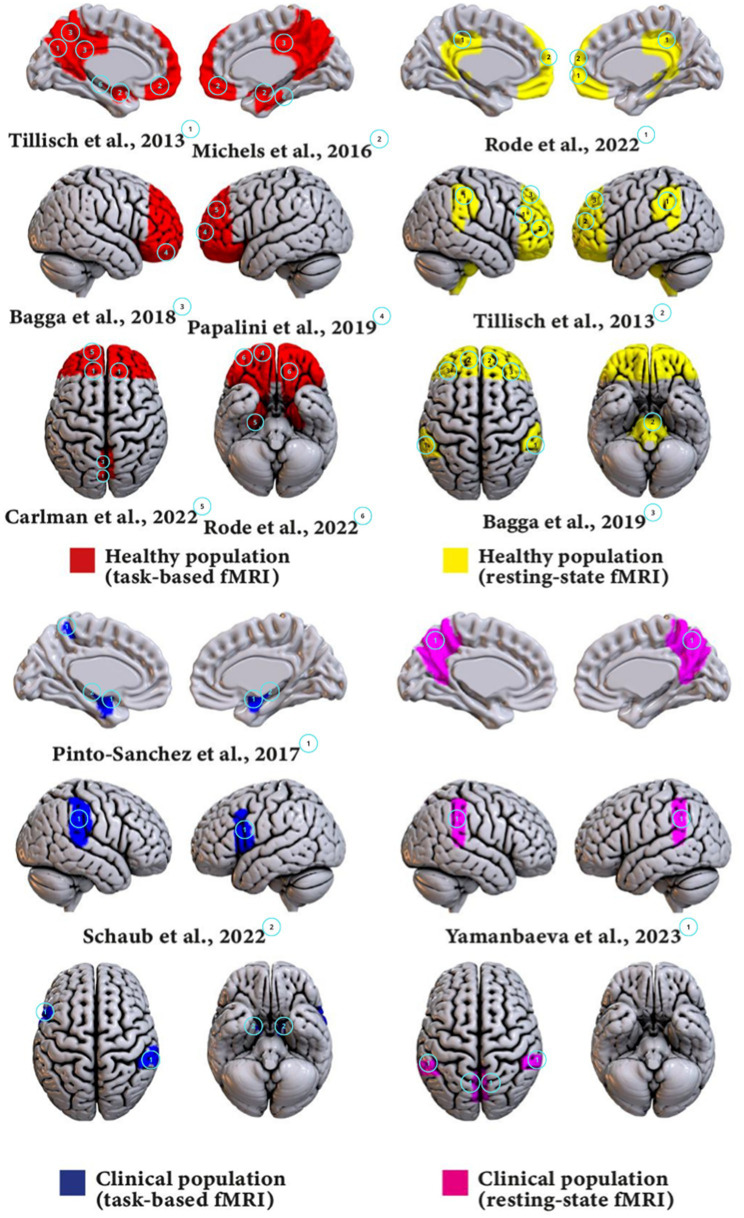
Visualizations of main findings for each group.

The studies included in this review examined the effects of probiotics on brain activity and functioning using a variety of fMRI methodologies, populations, and probiotic strains. These characteristics and the main results of each studies are detailed in Table 2 and summarized below (see Figure 2).

**Table 2:** Characteristics of studies included in the review, detailing study populations, types of probiotic interventions, fMRI methodologies, and main findings.

Author (year)	Study population		Intervention		FMRI Methodology		Main results
	Healthy	Clinical^*^	Probiotic name	Bacterial strains	Task	Resting-state	
Bagga et al. (2018) (56)	X		Eco- logic^®^825	9 strains: *Lactobacillus casei* W56, Lacto- bacillus acidophilus W22, *Lactobacillus paracasei* W20, *Bifidobacterium lactis* W51, *Lactobacillus salivarius* W24, *Lactococcus lactis* W19, *Bifidobacterium lactis* W52, Lacto- bacillus plantarum W62 and *Bifidobacterium bifidum* W23	X		Decreased activity in the precuneus, mid cingulum and the parahippocampal gyrus, lingual gyrus and calcarine gyrus Increased activity in the posterior cingulum
Bagga et al. (2019) (51)	X		Eco- logic^®^825	9 strains: *Lactobacillus casei* W56, Lacto- bacillus acidophilus W22, *Lactobacillus paracasei* W20, *Bifidobacterium lactis* W51, *Lactobacillus salivarius* W24, *Lactococcus lactis* W19, *Bifidobacterium lactis* W52, Lacto- bacillus plantarum W62 and *Bifidobacterium bifidum* W23		X	Increased connectivity in SN, Decreased connectivity in DMN, Decreased connectivity in MFGN
Carlman et al. (2022) (79)	X		/	3 strains: *Bifidobacterium longum* R0175, *Lactobacillus helveticus* R0052 and Lactiplantibacillus plantarum R1012	X		Decreased activation in lateral orbital and ventral cingulate gyri Increased functional connectivity between the upper limbic region and medioventral area (fusiform gyrus)
Michels et al. (2016) (80)	X		/	/	X		Decreased activity in the amygdala and frontal cortex Increased activity in the IFG**
Papalini et al. (2019) (57)	X		Ecologic^®^Barrier	9 strains: *Bifidobacterium bifidum* W23, *Bifidobacterium lactis* W51, *Bifidobacterium lactis* W52, *Lactobacillus acidophilus* W37, *Lactobacillus brevis* W63, *Lactobacillus casei* W56, *Lactobacillus salivarius* W24, *Lactococcus lactis* W19 and *Lactococcus lactis* W58	X		Decreased activity in the prefrontal cortex
Pinto-Sanchez et al. (2017) (101)		X (IBS)	/	1 strain: *Bifidobacterium longum* NCC3001	X		Decreased activity in the amygdala, frontal and temporal cortices Increased activity in the occipital regions
Rode et al. (2022) (73)	X		Puraflor	3 strains: *Bifidobacterium longum* R0175, *Lactobacillus helveticus* R0052 and Lactiplantibacillus plantarum R1012	X		Increased activity in the OFC, Decreased connectivity between sub-clusters: frontal pole, superior temporal sulcus, caudal areas (lingual gyrus), occipital polar cortex, inferior frontal sulcus
Rode et al. (2022) (73)	X		Puraflor	3 strains: *Bifidobacterium longum* R0175, *Lactobacillus helveticus* R0052 and Lactiplantibacillus plantarum R1012		X	Increased connectivity of DMN, Decreased connectivity in the SN, frontoparietal and middle frontal/precentral gyri
Schaub et al. (2022) (102)		X (MDD)	Vivomixx^®^	8 strains: *Streptococcus thermophilus* NCIMB 30438, *Bifidobacterium breve* NCIMB 30441, *Bifidobacterium longum* NCIMB 30435, *Bifidobacterium infantis* NCIMB 30436, Lactoba- cillus acidophilus NCIMB 30442, *Lactobacillus plantarum* NCIMB 30437, *Lactobacillus paracasei* NCIMB 30439, *Lactobacillus delbrueckii* subsp. Bulgaricus NCIMB 30440	X		Decreased functional activity in the putamen
Tillisch et al. (2013) (58)	X		Fermented milk product with probiotic (FMPP)	*Bifidobacterium animalis* subsp. Lactis, Streptococcus thermophiles, *Lactobacillus bulgaricus* and *Lactococcus lactis* subsp. Lactis	X	X	Decreased activity in the primary viscerosensory and somatosensory cortices, amygdala and precuneus Changes in PAG
Yamanbaeva et al. (2023) (103)		X (MDD)	Vivomixx^®^	8 strains: *Streptococcus thermophilus* NCIMB 30438, Bifidobacterium breve NCIMB 30441, *Bifidobacterium longum* NCIMB 30435, *Bifidobacterium infantis* NCIMB 30436, *Lactobacillus acidophilus* NCIMB 30442, Lactobacillus plantarum NCIMB 30437, *Lactobacillus paracasei* NCIMB 30439, *Lactobacillus delbrueckii* subsp. Bulgaricus NCIMB 30440		X	Increased connectivity of precuneus with: subcallosal cortex, amygdala, hippocampus and left temporal pole Decreased connectivity of the left superior parietal pole with: subcallosal cortex, right amygdala and left hippocampus; but increased with the OFC

*The clinical population consists exclusively of patients that were randomly assigned to either the probiotic group or the placebo group. No healthy control participants were included in this clinical population assessment. **uncorrected results. SN, salience network; DMN, default mode network; MFGN, middle and superior frontal gyrus network; PAG, periaqueductal gray; OFC; orbito-frontal cortex; IFG, inferior frontal gyrus; IBS, irritable bowel syndrome; MDD, major depressive disorder.

The studies involved both healthy individuals and clinical populations with specific conditions such as Major Depressive Disorder (MDD) and Irritable Bowel Syndrome (IBS).

Note that the interventions varied significantly in terms of the type and number of probiotic strains used. Multi-strain probiotics include a combination of different bacterial species, while single-strain probiotics involve a single bacterial species.

The fMRI methodologies employed in these studies included both task-based and resting-state fMRI. Task-based fMRI involves participants performing specific tasks while brain activity is measured, providing insights into how probiotics might affect brain function during cognitive and emotional challenges. Resting-state fMRI measures brain activity while participants are at rest, offering a view into the brain’s intrinsic functional connectivity and how it might be modulated by probiotic intake.

### 3.2 Main findings

#### 3.2.1 Healthy population

##### 3.2.1.1 Task-based fMRI

In healthy populations, task-based fMRI studies provide compelling evidence that four weeks probiotic supplementation appears to modulate brain function. In particular, the results from these task-fMRI studies taken into consideration seem to suggest the alteration of emotional regulation, sensory processing and cognitive control.

Specifically, Tillisch et al. (58) found that the intake of a fermented milk product with probiotics (*Bifidobacterium animalis subsp Lactis, Streptococcus thermophiles, Lactobacillus bulgaricus, and Lactococcus lactis subsp Lactis*) was associated with reduced activation in brain regions involved in emotion processing, suggesting an attenuation of the neural response to emotional stimuli. These reductions in brain activity were found in somatosensory cortices, precuneus, and amygdala. This influence on the sensory and emotional brain networks may underpin some of the behavioral changes observed with probiotic supplementation, such as improvements in mood and cognitive function (5, 65, 66). Such effects could be particularly beneficial in the context of mental health conditions like anxiety and depression, where these brain regions are often implicated (5, 65–67).

Bagga et al. (56) expanded on these findings by demonstrating changes in brain activation patterns during emotional decision-making and recognition memory tasks, indicating the potential of probiotics (specifically Ecologic^®^825) to influence emotional processing. Reduced activity in regions like the precuneus, middle cingulate cortex, and parahippocampal gyrus suggests decreased engagement in self-referential thoughts or stress, possibly enhancing focus on external tasks (68–70). More over, increased activity in the posterior cingulate cortex might enhance memory processing and integration, compensating for decreased activity in other previous areas involved in the default mode network (71, 72).

Always in the context of emotional processing, Rode et al. (73) recently reported increased activation in the orbitofrontal cortex (OFC) following probiotic supplementation with Puraflor. This suggests enhanced capacity or efficiency in processing emotional information, potentially leading to better emotional regulation and decision-making under emotional contexts (74–76). The study highlights also reduced connectivity between critical brain areas such as the frontal pole and superior temporal sulcus. This could indicate a more efficient neural response that reduces sensitivity to negative stimuli, which could help dampen adverse emotional reactions in conditions like anxiety or depression (77, 78). This, combined with increased activity in the OFC, could indicate a modulation of the brain’s emotional circuitry towards greater efficiency.

The studies by Carlman et al. (79) and Papalini et al. (57) highlight the potential of probiotics in modulating brain functions related to emotional, stress, and cognitive processes. In details, Carlman et al. (79) observed that a specific probiotic mixture, containing *Bifidobacterium longum R0175, Lactobacillus helveticus R0052 and Lactiplantibacillus plantarum R1012*, reduced activation in key emotional regulation areas such as the lateral orbital and ventral cingulate gyri and increased connectivity in regions like the upper limbic and fusiform gyrus. This suggests that probiotics can alter brain activity and connectivity to modulate responses to stress, with a direct impact on neural pathways independent of hormonal stress response mechanisms. Meanwhile, Papalini et al. (57) demonstrated that a multi-species probiotic (Ecologic^®^Barrier) could buffer the detrimental effects of acute stress on working memory, specifically showing decreased recruitment of the prefrontal cortex during stressful cognitive tasks in the probiotic group. This could imply a more efficient neural response, allowing for maintained or improved cognitive performance under stress.

Finally, Michels et al. (80) involved tasks like the Emotional Face Matching Paradigm and Emotional Stroop Task in its fMRI study but yielded no significant results possibly due to a small sample size, making it difficult to draw definitive conclusions. However, exploratory analyses using uncorrected thresholds showed noteworthy brain activity changes, such as decreased activity in the amygdala during the Emotional Stroop Task, suggesting improved emotional regulation (81), and increased activity in the inferior frontal gyrus and ventromedial prefrontal cortex during the Classic Color-Word Stroop Task, indicating enhanced cognitive control (82, 83). These preliminary findings propose that probiotics may influence brain function to better manage emotional and cognitive conflicts, although these should be cautiously interpreted given the limitations of the study.

These task-fMRI studies on healthy population highlight a significant theme: probiotics could modulate brain function in healthy individuals by affecting regions critical for emotional regulation, sensory processing, and cognitive performance.

##### 3.2.1.2 Resting-state fMRI

Expanding upon the interesting insights obtained from task-based fMRI studies on healthy populations, rs-fMRI offers a distinct but complementary perspective on the brain’s intrinsic functional connectivity altered by probiotic supplementation. This method offers a distinct perspective for observing the baseline functional architecture of the brain (84, 85), revealing the subtle yet significant impacts of probiotics on brain networks without the need of specific tasks or stimuli.

In the study by Bagga et al. (51), rs-fMRI revealed notable changes in key large-scale functional networks following administration of Ecologic^®^825. The authors found an increased activation within the Salience Network (SN), particularly in the cingulate gyrus. This area plays a crucial role in integrating sensory information and is pivotal for attention and emotional processing (86, 87). This suggests that probiotics might enhance the ability of the brain to prioritize and respond to salient stimuli, an integral function to adaptive behavior and cognitive processing. Conversely, a decrease in activation was observed in the Default Mode Network (DMN), especially in the frontal pole and superior frontal gyrus, alongside the paracingulate gyrus. The DMN is typically active during rest and is involved in self-referential thoughts and mind-wandering (88–90); thus, reduced activation may indicate a shift towards a more outwardly focused cognitive state, potentially reducing distractibility and enhancing task-focused attention (91, 92).

Further, Tillisch et al. (58) also utilized rs-fMRI to explore the influence of Fermented milk product with probiotic (FMPP) on brain function, particularly focusing on the periaqueductal gray (PAG)—a region pivotal in integrating interoceptive, affective, and prefrontal regions (93). Modifications in the PAG could influence the brain’s response to pain and emotional stimuli, reflecting a potential mechanism through which probiotics could modulate pain perception and emotional regulation. This finding complements the task-based observations of enhanced emotional regulation and cognitive control, suggesting that probiotics could contribute to a more resilient neural framework against nociceptive and emotional disturbances.

However, contrasting results emerge in later studies, such as those by Rode et al. (73), which administered a probiotic supplementation with Puraflor (*Bifidobacterium longum R0175, Lactobacillus helveticus R0052 and Lactiplantibacillus plantarum R1012*) and documented changes in connectivity within and across brain networks. Enhancements in the DMN connectivity, particularly linking the postcentral gyrus and superior parietal lobule, might reflect an improved internal state of mind that could contribute to better memory and self-referential thought processes (94, 95). Additionally, increased connectivity between language networks and areas involved in language processing and visual recognition suggests that probiotics might also enhance cognitive functions related to communication and visual processing. At the same time, reductions in connectivity within the Salience and Frontoparietal Networks indicate a more streamlined brain function, possibly reflecting an optimized allocation of cognitive resources which is beneficial for both cognitive and emotional regulation (96, 97).

The discrepancies between Bagga et al. (51) and Rode et al. (73) findings in the DMN and SN could be attributed to differences in study methodologies, probiotic strains used, or individual variations in the gut–brain axis among participants. These inconsistencies underscore the complexity of neural responses to probiotics and the need for further research. However, despite the apparent inconsistency, both studies show functional alterations in areas that are part of these two fundamental networks.

These studies collectively emphasize a broader narrative: probiotic supplementation appears to modulate the resting brain function in a manner that could enhance cognitive efficiency, emotional stability, and behavioral performance.

#### 3.2.2 Clinical populations

Building upon findings from studies on healthy populations, which have showed the potential of probiotics to modulate brain function, this paragraph shifts focus towards clinical populations to deepen the understanding of the gut–brain axis. Specifically, it explores the impact of probiotics on individuals with Major Depressive Disorder (MDD) and Irritable Bowel Syndrome (IBS), examining how these microorganisms may influence neural pathways and emotional processing in these conditions. By contrasting these findings with those from healthy cohorts, where probiotics have been shown to enhance cognitive functions and emotional regulation, the aim is to uncover whether similar or distinct neural adaptations occur in response to probiotic supplementation among those with psychiatric or gastrointestinal disorders.

##### 3.2.2.1 Task-based fMRI

In their comprehensive study, Schaub et al. (102) explored the impact of probiotics, administering Vivomixx^®^ (see Table 2 for the specific strains), on individuals with MDD by using task-based fMRI to assess brain responses to emotional and neutral facial expressions. The study observed a significant reduction in activation of the putamen during the processing of neutral faces in the probiotic group compared to the placebo group. The putamen, integral to the emotional and reward processing circuits, typically exhibits dysregulated activity in MDD (98, 99). This dysregulation often manifests as heightened responses to negative stimuli and diminished responses to positive or neutral stimuli, contributing to the pervasive negative emotional bias in depression (100). The normalization of putamen activity suggests that probiotics could help recalibrate emotional processing, potentially reducing the negative bias and improving mood regulation in MDD patients.

Moreover, the study by Pinto-Sanchez et al. (101) investigated the effects of probiotics (*Bifidobacterium longum NCC3001*) on brain function in individuals with IBS. Utilizing task-based fMRI with a fear-inducing backward masking paradigm, the study showed distinct changes in brain activation patterns. Specifically, there was decreased activation in critical emotional processing areas such as the amygdala, frontal, and temporal cortices, regions known for their roles in fear and anxiety regulation (104). Conversely, an increase in activation was noted in the occipital regions, such as cuneus and middle occipital gyrus, which are primarily involved in visual processing. This shift in neural activation could suggest a rerouting of neural resources from emotional to perceptual processing areas in response to probiotics. Moreover, the study highlighted the potential neurochemical pathways influenced by probiotics. Notably, the increase in hippocampal Brain-Derived Neurotrophic Factor (BDNF) suggests a possible enhancement in neuroplasticity and neuronal health, which is often compromised in depression-related disorders (105, 106). Additionally, changes in the dopamine/noradrenaline pathway could be pivotal in mediating the mood-stabilizing effects of probiotics.

##### 3.2.2.2 Resting-state fMRI

Integrating rs-fMRI findings, Yamanbaeva et al. (103) report functional brain changes in patients with depression following probiotic supplementation with Vivomixx^®^ (see Table 2 for the specific strains). Increased connectivity between the precuneus and important brain nodes of the human connectome such as the subcallosal cortex, amygdala, hippocampus, and left temporal pole was observed, along with changes in connectivity patterns within the superior parietal lobule. These changes suggest a reorganization of pathways that underpin cognitive-emotion interactions, potentially contributing to cognitive improvements and symptom reduction in depression.

Collectively, these findings indicate that probiotics might offer neuroprotective benefits, influence emotional and cognitive regulation through their impact on brain network dynamics, and normalize brain functions typically dysregulated in depression. The evidence adds a critical dimension to the understanding of the gut–brain axis’s role in treating psychiatric disorders, underscoring the need for further research to elucidate the mechanisms by which probiotics exert these effects and to explore their therapeutic potential in larger, more diverse populations.

## 4 Discussion

This systematic review aimed to investigate the impact of probiotics on brain function, utilizing findings from both task-based and resting-state functional MRI (fMRI) studies across healthy and clinical populations. The collected evidence suggests that probiotics have a notable impact on functional connectivity with implications for both general cognitive functions and specific psychiatric conditions.

In healthy individuals, task-based fMRI studies (56–58, 73, 79, 80) demonstrated that probiotics could modulate brain activity related to emotional and stress processing. For instance, reduced activation in areas like the amygdala and the precuneus, and enhanced performance in cognitive tasks under stress were noted, suggesting that probiotics may help in dampening stress responses and improving cognitive efficiency under challenging conditions. Complementing these task-based observations, resting-state studies revealed (51, 58, 107) alterations in brain connectivity that promote cognitive and emotional stability, such as enhanced connectivity within the Salience Network and reduced activation in the Default Mode Network. These changes suggest a shift towards more efficient and externally focused brain states, which may underlie the cognitive and emotional benefits observed in the task-based studies.

In populations with specific clinical conditions, such as MDD and IBS, the effects of probiotics appear interesting. For MDD, the implications are particularly significant in terms of modifying dysfunctional emotional processing, while in IBS, the effects of probiotics may extend beyond gastrointestinal symptoms to influence emotional well-being and neurobiological responses to stress. Resting-state fMRI studies further supported these findings, with evidence from patients suffering for depression showing that probiotics could enhance connectivity in mood-regulating brain nodes, such as the precuneus.

Moreover, the reviewed studies collectively reveal that probiotics influence brain function through several interconnected mechanisms. One primary mechanism is the normalization of activity within neural networks that are typically dysregulated in psychiatric and neurological conditions. For instance, probiotics appear to modulate the Salience and Default Mode Networks, which are crucial for processing and filtering relevant stimuli and for self-referential mental activities, respectively. Dysregulation in these networks is often observed in conditions such as depression and anxiety (108, 109), suggesting that probiotics could play a significant role in restoring their normal function and thereby improving mental health outcomes. In addition to normalizing neural network activity, probiotics also demonstrate neuroprotective effects that might be mediated through multiple pathways. These effects include the stabilization of brain structures and the enhancement of neural connectivity, which could be partially attributed to the anti-inflammatory actions of probiotics (110–112). By reducing systemic and brain inflammation, probiotics might protect neuronal health and prevent or slow the progression of neurodegenerative processes (113–115). This protective mechanism is especially significant considering the growing evidence linking inflammation to various psychiatric and neurological disorders. Furthermore, probiotics influence areas of the brain involved in neural plasticity, such as the hippocampus and amygdala. These regions are essential for the brain’s ability to adapt structurally and functionally in response to environmental demands, stress, and learning processes (116–118). By modulating the activity and connectivity of these areas, probiotics may enhance the brain plasticity, facilitating better cognitive functions and emotional resilience. This modulation is likely facilitated by the production of neurotrophic factors, which are proteins that help to support the growth, survival, and differentiation of neurons. Collectively, these mechanisms suggest a comprehensive model wherein probiotics could significantly influence brain health by restoring balance to critical neural networks, protecting against neuroinflammation, and enhancing the adaptive capacities of the brain. This multifaceted impact highlights the potential of probiotics as a therapeutic tool for a range of psychiatric and neurological conditions, emphasizing the importance of further research into their specific effects and the optimal conditions for their use.

## 5 Open issues in the study of the gut–brain axis

The first constraint within the current review is the limited numbers of studies examining the impact of probiotics on brain functioning. This paucity of research restricts the ability to draw robust conclusions and completely comprehend the extent of probiotics’ influence on neuronal activity. The limited research base, which exclusively examines MDD and IBS, also constrains opportunities for replicating findings, a critical step in confirming results and establishing a reliable foundation for clinical applications. Increasing the size and range of studies would provide a more thorough comprehension of the impacts of probiotics on different neural systems.

### 5.1 The choice of emotional response tasks

Another issue in the reviewed studies involves the predetermined selection of tasks based on emotional responses, which might only reveal already established brain networks related to emotional processing. This emphasis can conceal other possible brain effects of probiotics that are not triggered by these tasks. However, the rs-fMRI approach offers a strategic alternative by evaluating brain activity in a spontaneous state, potentially revealing broader neural connectivity changes influenced by probiotics. Notably, a limited number of studies (51, 56, 58, 73, 107) have effectively used both task-fMRI and rs-fMRI to explore the gut–brain interaction, providing a more comprehensive view of how probiotics may affect brain function.

Despite these limitations, emotional tasks may have been selected for these studies for their ability to activate crucial brain regions like the amygdala, prefrontal cortex, and hippocampus, areas significantly affected by gut microbiota as evidenced by previous studies (119, 120). These regions have been shown to be highly involved in the symptomatology of mental health conditions such as anxiety, sadness, schizophrenia, autism, and moderate cognitive impairment, all of which include difficulties in regulating emotions and have been associated with imbalances in the gut (17–19). These tasks rely on the gut–brain axis and involve hormonal, immunological, and cerebral pathways. They are also affected by neurotransmitter systems such as serotonin and dopamine, which are strictly related with gut health (121–124). Thus, the use of emotional tasks allows for the exploration of how gut health impacts emotional regulation and mental health.

### 5.2 Probiotic types and dietary variations

This review also identifies a significant variability in the strains of probiotics used, their dosages, the duration of the interventions, and whether probiotics were administered alone or in conjunction with medications. These variations can potentially influence the outcomes and are critical for interpreting the effectiveness and applicability of probiotics. For example, the concurrent administration of medications may obscure whether observed neuronal effects are genuinely due to the probiotics or to a synergistic effect with the drug compound, complicating the interpretation of probiotic efficacy (125–127). It would be interesting to determine whether there is a dose-dependent or type-dependent relationship between probiotic supplements and changes in brain function. Additionally, dietary variations among participants recruited in the studies can significantly affect gut microbiota, potentially influencing the gut–brain axis and, consequently, the outcomes of interventions involving probiotics (128–130). Furthermore, the assumption that prebiotics may help ameliorate the effects of probiotics warrants consideration. Prebiotics, which stimulate the growth and activity of beneficial bacteria, could enhance the survival and efficacy of probiotics, leading to more pronounced effects on brain function and overall health. Studies have shown that prebiotics can improve the gut environment, making it more conducive for probiotics to thrive and exert their beneficial effects (1, 2, 130). This synergistic relationship suggests that the combined use of probiotics and prebiotics could be more effective than probiotics alone, further emphasizing the need to control for dietary factors in probiotic research.

## 6 Conclusion and future perspective

This systematic review highlights the significant impact of probiotics on the gut–brain axis, as evidenced by neuroimaging studies. Probiotics demonstrate the potential to modulate brain function and connectivity, particularly in regions involved in emotional regulation, sensory processing, and cognitive control. In specific clinical conditions, namely major depressive disorder (MDD) and irritable bowel syndrome (IBS), probiotics seem to normalize brain activity and improve mood-regulating networks, suggesting their potential as therapeutic agents. Despite these promising findings, methodological variability and limited sample sizes underline the need for more stringent experimental designs and longer-term studies.

To address these gaps, future research should employ rigorous experimental designs with well-controlled variables. This includes stabilizing dietary intake among participants, using detailed food diaries, and conducting follow-ups at multiple time points to observe changes over time and potential decay in the effects of probiotics. Studies like those by Pinto-Sanchez et al. (101) and Rode et al. (73) exemplify the benefits of such approaches by including follow-up assessments that provide insights into the temporal dynamics of probiotic effects. Further, despite promising initial findings on probiotics effects on brain function, several critical areas require deeper exploration to improve our understanding and clinical application of probiotics. Longitudinal studies are essential to determine the longevity of probiotics effects also to possibly guide clinical usage and recommendations. Research must also be extended to a broader range of demographic groups to ensure that findings are generalizable across different ages, ethnicities, and health conditions. Moreover, more detailed mechanistic studies are necessary to elucidate the pathways through which probiotics influence the brain, potentially incorporating techniques from metabolomics and microbiomics to uncover underlying biochemical interactions (12, 131, 132).

## Data Availability

The original contributions presented in the study are included in the article/Supplementary material, further inquiries can be directed to the corresponding author.
