# Supplementary material for: From gut to brain: unveiling probiotic effects through a neuroimaging perspective—A systematic review of randomized controlled trials
Source: Front Nutr. 2024 Sep 18;11:1446854. doi: 10.3389/fnut.2024.1446854 (PMC11444994; doi:10.3389/fnut.2024.1446854)
Supplement: Supplementary file 1 [file Table_1.DOCX]

Supplementary Material

| **Section A: Is the basic study design valid for a RCT?** |  |
| --- | --- |
| 1. Whether the study addressed a clearly focused issue  2. Whether the assignment of patients to treatments was randomized.  3. Whether all patients who entered the trial were properly accounted for at its conclusion. | |
| **Section B: Was the study methodologically sound?** | |
| 4. Whether patients, health workers, and study personnel were ‘blind’ to treatment.  5. Whether the groups were similar at the start of the trial.  6. Whether, aside from the experimental intervention, the groups were treated equally. | |
| **Section C: What are the results?** | |
| 7. Whether the effects of the intervention were reported comprehensively.  8. Whether the precision of the estimate of the treatment effect was reported.  9. Whether the benefits of the intervention outweigh the harms and costs. | |
| **Section D: Will the results help locally?** | |
| 10. Whether the results can be applied to the local population  11. Whether the experimental intervention provides greater value than existing interventions. | |
|  | |

CASP Checklist

To assess the quality of the included studies, we utilized the Critical Appraisal Skills Programme (CASP) checklist for Randomized Controlled Trials (RCTs). The CASP checklist is a standardized tool that evaluates the methodological quality of studies based on 11 key questions. These questions address the validity of the study design, the precision of the results, and the applicability of the findings to the local population. For each included study, the following aspects were assessed and reported in Table S2.

**Table S1**

**Table S2**

| **AUTHOR**  **(YEAR)** | **Section A** | | | **Section B** | | | **Section C** | | | **Section D** | |
| --- | --- | --- | --- | --- | --- | --- | --- | --- | --- | --- | --- |
|  | **Clear Focus** | **Randomization** | **Accounting** | **Blinding** | **Baseline Similarity** | **Equal Treatment** | **Effect  Reported** | **Precision  Reported** | **Cost-benefit** | **Local applicability** | **Value of intervention** |
| Bagga et al. (2018) | Yes | Yes | Yes | Yes | Yes | Yes | Yes | Yes | Yes | Yes | Can't tell |
| Bagga et al. (2019) | Yes | Yes | Yes | Yes | Yes | Yes | Yes | Yes | Yes | Yes | Can't tell |
| Carlman et al. (2022) | Yes | Yes | Yes | Yes | Yes | Yes | Yes | Yes | Yes | Yes | Can't tell |
| Michels et al. (2016) | Yes | Yes | Yes | Yes | Yes | Yes | Yes | Yes | Yes | Yes | Can't tell |
| Papalini et al. (2019) | Yes | Yes | Yes | Yes | Yes | Yes | Yes | Yes | Yes | Yes | Can't tell |
| Pinto-Sanchez et al. (2017) | Yes | Yes | Yes | Yes | Yes | Yes | Yes | Yes | Yes | Yes | Can't tell |
| Rode et al. (2022) | Yes | Yes | Yes | Yes | Yes | Yes | Yes | Yes | Yes | Yes | Can't tell |
| Rode et al. (2022) | Yes | Yes | Yes | Yes | Yes | Yes | Yes | Yes | Yes | Yes | Can't tell |
| Schaub et al. (2022) | Yes | Yes | Yes | Yes | Yes | Yes | Yes | Yes | Yes | Yes | Can't tell |
| Tillisch et al. (2013) | Yes | Yes | Yes | Yes | Yes | Yes | Yes | Yes | Yes | Yes | Can't tell |
| Yamanbaeva et al. (2023) | Yes | Yes | Yes | Yes | Yes | Yes | Yes | Yes | Yes | Yes | Can't tell |
